# Lipidomics of facial sebum in the comparison between acne and non-acne adolescents with dark skin

**DOI:** 10.1038/s41598-021-96043-x

**Published:** 2021-08-16

**Authors:** Obumneme Emeka Okoro, Adebomi Adenle, Matteo Ludovici, Mauro Truglio, Federico Marini, Emanuela Camera

**Affiliations:** 1 Dermatology Unit, Federal Medical Centre, Keffi, Nasarawa Nigeria; 2 Laboratory of Cutaneous Physiopathology, San Gallicano Dermatological Institute-IRCCS, Rome, Italy; 3 Department of Chemistry, University of Rome ‘La Sapienza’, Rome, Italy

**Keywords:** Data mining, Data processing, Lipidomics, Biomarkers

## Abstract

Lipidomics is advantageous in the study of sebum perturbations occurring in acne. An extended evaluation of the sebum lipid profiles in acne-prone sebaceous areas is lacking in dark skin. Yet, there is a void space in understanding how the building blocks of sebum lipids, i.e. individual fatty acids (FAs), are intertwined with acne-prone skin. We aimed to determine the sebum lipidome in facial areas of adolescents with and without acne in Nigeria. A cross-sectional analytical study was conducted in 60 adolescents/young adults divided in 30 acne patients (15F, 15M) and 30 age and sex-matched controls. Sebum samples obtained from foreheads and cheeks were analysed separately by gas chromatography–mass spectrometry (GCMS) and thin layer chromatography (HPTLC). Distributions of sebum components were investigated with multivariate ANOVA-simultaneous component analysis (ASCA). Sebum incretion in acne was paralleled by significantly higher abundance of triglycerides, wax esters, and squalene together with monounsaturated FAs (MUFAs), and straight chain saturated FAs (SFAs), especially those with odd-carbon chain, i.e. C13:0, C15:0, and C17:0. Profiling weight/weight percentage of individual components revealed that, in acne, the free FAs (FFAs) array was shifted towards higher relative abundance of the SFAs C15:0, C16:0, and C17:0 and lower percentage of the anteiso-branched FFAs with 12, 14, 16, and 18 carbons. In acne patients, MUFAs and PUFAs were quantitatively increased and decreased on foreheads and cheeks, respectively. Relative abundance of fatty alcohols was decreased in acne independent on the site. The results indicated that acne associates with site-specific derangement of the pathways regulating the balance among odd straight-chain and branched-chain SFAs, MUFAs, which included sapienate (C16:1n-10), PUFAs, and squalene.

## Introduction

Acne vulgaris is a chronic disorder of the pilosebaceous unit characterized by hyperseborrhea, dyseborrhea, skin dysbiosis, hyperkeratinisation, and inflammation, which altogether concur to the development of acne lesions^1^. The prevalence among adolescents varies between 30 and 90% in various population groups and geographical areas in the world^2–5^. Acne is a common reason for dermatological consult among adolescents with dark skin^6^. Increased sebum secretion is one of the multiple factors involved in the pathogenesis of acne. Positive correlations exist between sebum secretion rates and lesion counts^7–11^. Derangement in the lipid composition is an important contributor to the pathogenicity of sebum. Deficit of linoleic acid^12^, increase of overall percentage of free fatty acids (FFAs) and promotion of squalene peroxidation^13–15^, are accounted among the triggers for acne. The unbalanced triglycerides (TGs) to wax esters (WEs) ratio^16,17^ and higher levels of monounsaturated fatty acids (MUFAs)^18^ characterize acne sebum. Pappas et al.^19^ reported that squalene levels were higher in acne patients while our group reported higher levels of diglycerides (DGs) in sebum of adolescents with acne^20^. Density of sebaceous glands (SGs) and hormone receptors, together with gender, likely contribute to the different patterns of acne. Sex-related differences in the skin surface lipids composition have been observed in some cases and not observed in other ones^21–23^. Skin lipid composition is broadly shared among White, Asian, and African ethnicities^22,24,25^. However, there is a gap in the knowledge on biological characteristics of sebum production in ethnic patients. Distribution of sebum on the face is uneven with higher levels in the T-zone (forehead-nose-chin) compared to the U-zone (cheekbones-cheeks-jaw). In keeping with previous evidence^11,26^, we have reported a correlation of sebum levels with acne lesions in the U-zone not existing in the T-zone among adolescents with acne in Nigeria^10^. These observations could be related to the variation in sebum composition in the facial zones. The complexity of sebum and the multiplicity of the sebogenic pathways potentially involved in acne call for an extended and simultaneous detection of sebum components. There are no studies that compared sebum components in different facial areas in skin of colour. Our aim was to determine the profiles of abundance of sebum lipids in acne patients and unaffected controls of both genders in Nigeria.

## Results

### Demographics and acne grading

Demographics and distribution of the counted lesions of acne patients are detailed in the Supplementary Table S1. The mean age (years) of the acne patients and of controls was 17.2 ± 1.76, and 17.5 ± 1.76, respectively. Among the affected group, 15 (50%) had mild acne while 10 (33.3%) and 5 (16.7%) had moderate and severe acne respectively.

### Determination of elemental components of facial sebum

Forty-one FFAs, and 7 FOHs were quantified by GCMS (Supplementary Table S2 and Supplementary Fig. S1). FFAs consisted of saturated and unsaturated species. Among saturated FFAs, several branched FFAs with one methyl branching at the iso (ibrFA) and the anteiso (abrFA) positions were chromatographically resolved from the corresponding straight chain isomer with the same number of carbon-atoms. FFAs were categorized as even (eFA) and odd (oFA) ones according to their even or odd number of carbon atoms. The ibrFAs, and abrFAs subgroups included 7 members each, with carbon atoms ranging from 12 to 18. The subclasses of eFAs, oFAs, MUFAs, and PUFAs included 8, 7, 10, and 2 members, respectively. The eFAs and oFAs together were SFAs with chain length ranging from 12 to 26 carbon atoms. MUFAs had mainly an even number of carbons, with the exception of C15:1, and C17:1. The latter one was found in both straight and branched isomers, which were assigned tentatively according to GCMS criteria. The C16:1 MUFA was characterized as sapienate (C16:1n-10) based on the same elution time and MS spectrum of the authentic reference compound and the resolution from palmitoleate (C16:1n-7), which eluted later. Palmitoleate was excluded from the quantitation due to its negligible amounts in sebum. The C18:1 MUFA appeared as a single peak. Likely, C18:1 isomers were not separated under the used GCMS conditions. FOHs with a chain length between 14 and 26 carbon atoms, were either confirmed with authentic compounds or tentatively assigned based on their MS spectrum and elution time (Supplementary Table S2). To visualize the within-class distributions of FFAs and FOHs, their amounts on foreheads and cheeks were averaged and plotted in radar diagrams (Supplementary Fig. S2). Polarization of FFAs and FOHs distribution was apparent in both NA and A groups. The top 3 members, ordered according to their abundance, were, in each subclass, as follows: 13Me-C15:0 > 14Me-C16:0 > 12Me-C14:0 for the ibrFAs; 12Me-C15:0 > 15Me-C18:0 > 13Me-C16:0 for the abrFAs; C16:0 > C18:0 >  > C14:0 for eFAs; C15:0 > C17:0 > C21:0 for the oFAs; C18:1 > C16:1 > C17:1 for the MUFAs, and FOH18:0 >  > FOH16:0 > FOH22:0 for the FOHs. The PUFA C18:2 was more abundant than C20:2.

**Figure 1:**
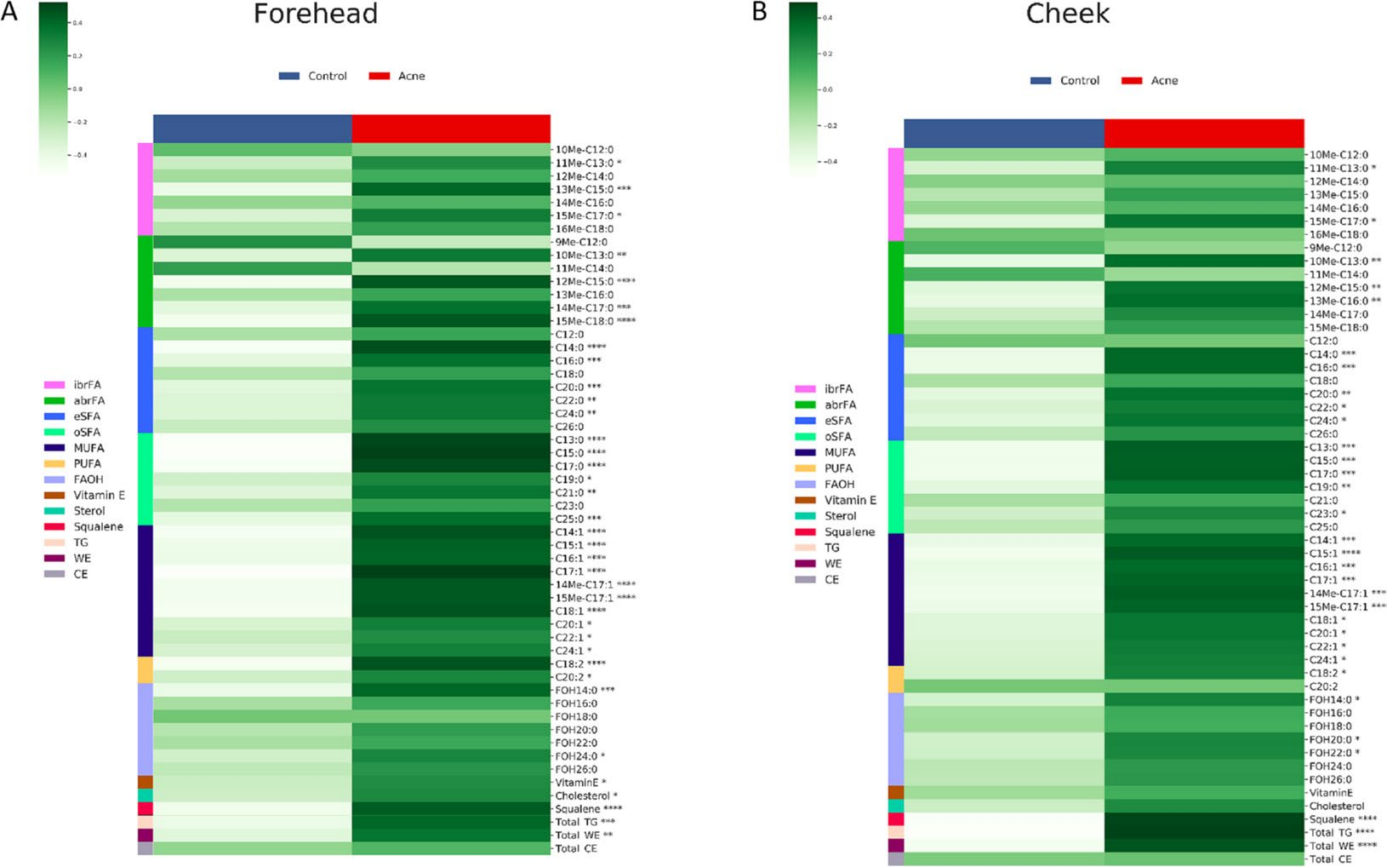
Heatmaps of amounts in µg of lipids quantified in sebum sampled with Sebutape patches on foreheads (**A**) and cheeks (**B**). Triglycerides (TGs), wax esters (WEs), and cholesterol esters (CEs) were quantified by HPTLC, whereas free fatty acids (FFAs), fatty alcohols (FOHs), squalene and cholesterol were quantified by GCMS. The total sebum amounts were obtained by summing the quantitative data obtained by HPTLC and GCMS. The heatmaps were generated by running custom Python code that uses the open source Matplotlib library. Significant differences were determined with Mann–Whitney test. *p ≤ 0.05, **p < 0.001, ***p < 0.005, ****p < 0.0001.

Separation of sebum lipids by HPTLC developed bands due to six major components, cholesterol, FFAs, TGs, WEs, CEs, and squalene (Supplementary Fig. S1), according to their elution order. Heat maps of the average amounts of sebum lipids in foreheads and cheeks in NA and A groups are reported in the Fig. 1A,B, respectively. It is apparent that the amounts of most sebum lipids were significantly elevated in the A group. Inspection of the abundance profiles revealed that specific FFAs and FOHs members were affected more than others in the two sites. The Supplementary Table S3 reports the amounts of sebum lipids (average µg ± SD) represented in the heat maps.

### Sebum excretion rates and correlation of sebum lipids with acne lesions

Sebum weights were obtained by summing the amounts (µg) of individual lipids quantified by GCMS and HPTLC and were used to obtain the sebum excretion rates (SER), which expresses the µg secreted on surface unit per minute (µg/cm^2^/min). SER values were significantly higher in acne patients (Supplementary Fig. S3) at both face sites, as shown by the p-values in the table included. SER values determined from cheeks were higher than those obtained from foreheads in the affected group only (p < 0.01). The associations of average composition of sebum from foreheads and cheeks with lesion counts was investigated by Spearman’s correlation. The amounts of squalene were positively correlated with the number of comedones (R = 0.47, p < 0.05, data not shown).

### ASCA analysis on the relative concentrations of individual lipids

**Figure 2:**
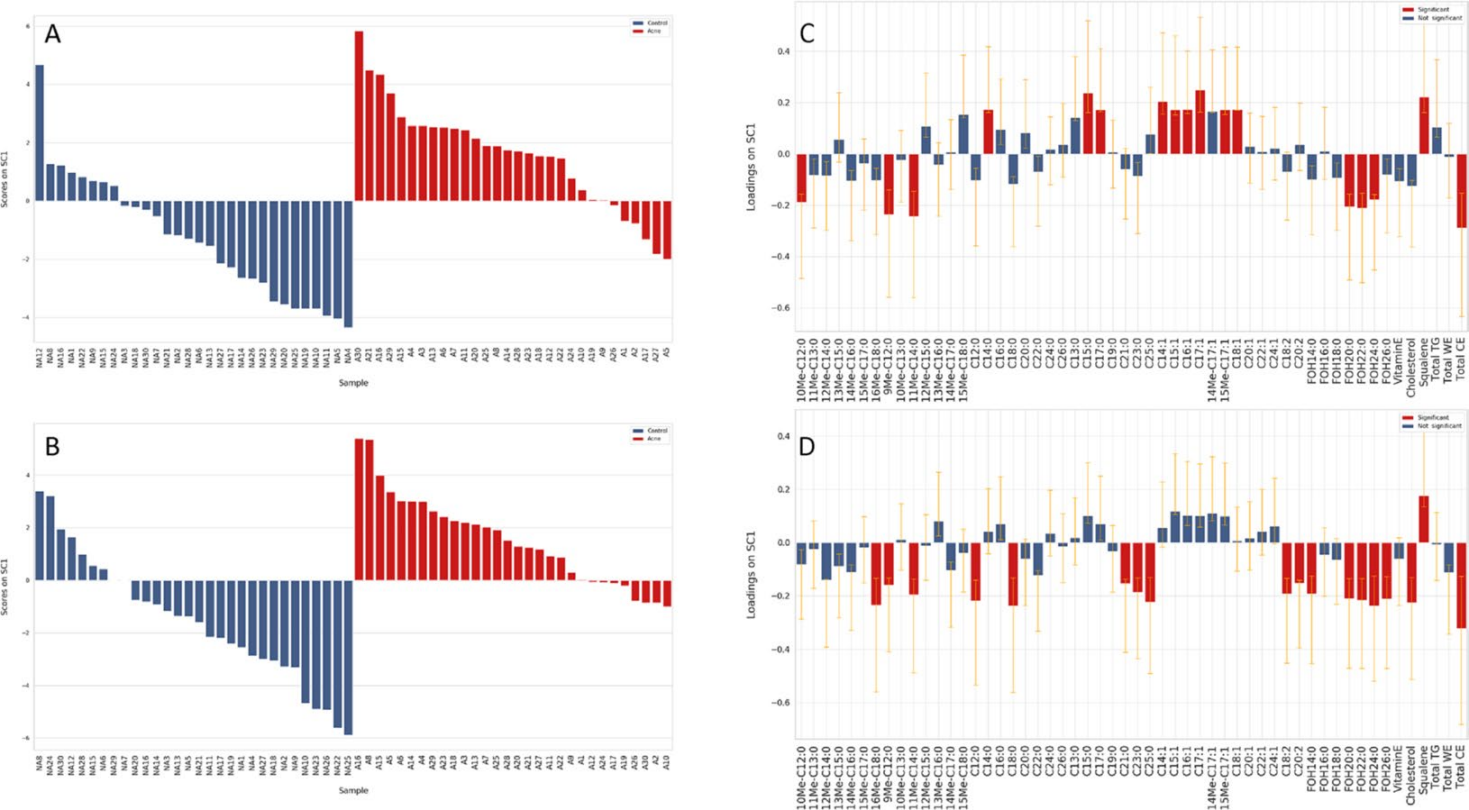
SCA analysis on the effect matrix (normalized quantities) for acne condition investigated in sebum from foreheads (**A**–**C**) and from cheeks (**B**–**D**). (**A**,**B**) Scores plots after projection of the residuals onto the space spanned by the only significant PC; legend: blue = non-acne; red = acne. (**A**) and (**B**) plots refer to the scores of foreheads and cheeks, respectively. (**C**,**D**) Variable loadings on SC1, together with their confidence interval (red and blue bars indicate significantly and not significantly contributing descriptors, respectively). (**C**) and (**D**) bar graphs refer to the loadings on the SC1 of individual lipids in foreheads and cheeks, respectively.

ASCA analysis, which is described in Appendix, was performed on the data set after normalization of the absolute individual amounts by the total sebum quantity (weight/weight percentage). The results of SCA analysis for the effect of the acne condition on the relative amounts on cheeks and foreheads are reported in Fig. 2. The percentage of two abrFFAs, i.e. 9Me-C12:0, and 11Me-C14:0, was lower on the forehead; whereas the ibrFFAs 10Me-C12:0, and 16Me-C18:0 had lower percentage on foreheads and cheeks, respectively. SFAs, and several MUFAs showed significantly higher percentage in the A group limited to the forehead. Percentages of short and long chain SFAs, PUFAs, and cholesterol, were decreased in A at the cheeks. Several FOHs were present at lower percentage in A, on both sites. Percentages of squalene and cholesterol were, respectively, higher and lower in the A group regardless the face site. Among the A subjects, outliers accounted for the 20% and 27% of the entire A group when scores were examined in the foreheads and the cheek lipid profiles, respectively. The comedone count (16.6 ± 4.2) in the outlier group was lower than the rest of the A subjects (24.4 ± 10.4), however the difference did not reach significance (p > 0.05). Among the 11 outliers for either forehead or cheek sites, 7 belonged to the mild cases; 3 and 1 to the moderate and the severe cases, respectively. The number of severe cases was insufficient to highlight differences due to the acne grade. Moreover, the severe cases presented scores that overlapped with those observed for the entire A group. Owing to the multiplicity of factors determining the severity of acne, including the type of lesions and patterns of distribution, a larger number of subjects is necessary to derive sebum signatures associated with severe grades.

## Discussion

This study is the first to analyse sebum composition among adolescents of African descent with and without acne using combined GCMS and HPTLC analyses and multivariate statistics. The size of the control and affected groups was consistent with that of studies on acne. The skin surface lipids from both foreheads and cheeks were dominated by sebaceous lipids, as demonstrated by the detection of components unique to sebum, i.e. squalene and WEs^17,27^. The calculated SER were consistent with our previous data based on sebum amounts determined gravimetrically on the sampling tapes^20^. The amounts of sebum components were unevenly distributed on the face of the acne group, with cheeks bearing more sebum than foreheads. This observation is in contrast with the casual sebum levels determined with the Sebumeter on the same sites and ethnic group with similar age and gender distribution^10^. The lack of studies comparing instrumental assessments with quantitative determination of sebum components limits conclusions^28^. The overall results demonstrated that the facial sebum secretion was higher in acne patients compared to age and gender matched controls, at both face sites, in keeping with established pathogenic factors of acne^10^. The higher amounts of sebum in acne were mirrored by significantly higher levels of individual lipids, i.e. squalene, cholesterol, and vitamin E, or lipid families, such as FFAs, FOHs, TGs, and WEs. Squalene levels correlated positively with the counts of non-inflammatory lesions, in agreement with the occurrence of squalene peroxide and the depletion of vitamin E in acne sebum^17,29,30^ and the patterns of acne distribution^11^. Among FFAs, most straight and branched chain FFAs showed significantly higher levels in acne sebum. Previous studies have focused on the bound FAs originated from sebum lipids separated by HPTLC^19,22,31,32^. FFAs have been evaluated in different ethnicities, including the African ones, by LCMS^25,33^. The LC methods commonly used do not resolve the separation of isobaric FFAs limiting the comparability of branched FFAs between acne patients and their controls. Pappas et al. reported a lower amount of FFAs in sebum of acne patients not specific to straight or branched chain FFAs^19,31,32^. We observed that several straight and branched chain FFAs (mostly with odd carbon-number, i.e. C13, C15, and C17) were among the upregulated species. Noteworthy, the abrFFAs were more affected than ibrFFAs. These differences were suggestive of abnormal synthesis of the odd-chain FFAs in acne. Opposite to other biofluids, branched chain FFAs are relatively abundant in sebum^34^. Multiple sources contribute to their level, such as de novo synthesis by the SG, metabolism of skin microbes, and diet^35,36^.

Canonical FA synthesis is initiated from acetyl-CoA. Propionic acid, which is produced either by microbial metabolism or amino acid catabolism^37^, and valeric acid, are the precursors of odd numbered straight chained C13:0, C15:0, C17:0, which were found among the most upregulated FFAs in acne. Branched FAs are synthesized from branched starters like short chain organic acids, and aminoacids. In particular, isobutyrate and isovalerate acid are precursors of ibrFAs with even and odd carbon number, respectively. Leucine and isoleucine are the precursors of ibrFFAs and abrFFA, respectively, mostly with 15 and 17 carbon atoms. Valine is the starter of the even-numbered ibrFAs^38^. Further studies are needed to understand the complex interconnections sebum FFAs establish with short chain organic acids, and amino acid metabolism.

The sebaceous-type desaturation pathways of FFAs appeared to be relevant in acne. Induction of MUFAs with sebum-type chain length of 14–17 carbon atoms was very significant at both sites. MUFAs with epidermal-type chain length, i.e. 18–24 carbon atoms, were induced at a lesser extent. Incretion was more pronounced at cheeks. The SG expresses both fatty acid desaturase 2 (FADS2) and stearoyl-CoA desaturase 1 (SCD-1). FADS2 is a ∆6 desaturase that converts palmitate into sapienate (C16:1n-10) following the insertion of a C–C double bond at the position 6. SCD-1 is a ∆9 desaturase showing substrate preference towards stearate, which is converted into oleate (C18:1n-9); however, it can also yield palmitoleate (C16:1n-7) from palmitate. In our approach, the prevalent sapienate isomer could be clearly discriminated from palmitoleate, which appeared to be present at negligible amounts in sebum. In contrast, assignments of C18:1 isomers was ambiguous. The current knowledge is rather limited to exclude that stearate undertakes both desaturation routes in the SG. Recent evidence has demonstrated that FADS2 inserts a ∆6 double bond in the branched and straight C17:0^39^. GCMS profiles showed peaks tentatively assigned as branched and straight C17:1 MUFAs. Enhanced MUFAs/SFAs ratio for C16, C17, and C18 chains in acne were suggestive of promotion of multiple desaturation pathways. Multivariate analysis by means of ASCA revealed that straight FFAs and branched FFAs with even carbon number were increased at a lesser extent than those with odd-chain length. One possibility is that the availability of precursors of even-chain branched FFAs can be limited compared to that of precursors of odd-chain branched FFAs. The lower weight/weight percentage of ibrFAs and abrFAs with even-chain in acne sebum might suggest that the de novo synthesis of these FFAs is left behind in competitive biosynthetic pathways leading to straight chain oFFAs and eFFAs. The profiles of relative abundance also revealed a shift towards higher MUFAs in acne, especially on foreheads. This evidence reinforces the involvement of the FADS2 pathway in the promotion of the sebum synthesis and occurrence of acne. Enhanced FADS2 activity leading to higher C16:1/C16:0 ratio has been implicated in the acnegenic effects of high glycaemic loads diets^18,40^. The lower levels of C18:2 found in acne sebum agrees with the deficit of linoleic acid reported in previous studies^12,41^. Profiles of abundance of C20:2 paralleled those observed for C18:2 suggesting that these two PUFAs are related metabolites. The higher levels of squalene and lower amounts of cholesterol in acne suggests a possible further inhibition of post-squalene cholesterol biosynthesis in the SG. For the first time, FOHs were examined for their impact on sebum composition in healthy and acne sebum. FOHs bind together with FAs to form WEs. Although pathways of FOHs and FFAs biosynthesis are intersected, the knowledge on the steps preceding the FOHs biosynthesis in the SG is rather limited^39^. The overall data are in favour of uneven distribution of sebum components on the face. The differences in the levels of sebum lipid components between foreheads and cheeks were more pronounced in acne patients. The ASCA approach identified outliers in both NA and A groups, highlighting the need to stratify cases and recognize patterns of acne-like sebum profiles in absence of clinical manifestations. The differences found between foreheads and cheeks may be related to distribution, size and activity of the SGs^42,43^ and may partly explain the patterns of inflammatory dermatoses on the T- and U-zones^10^. In particular, the data suggested a different impact of the disorder on multiple pathways leading to the FFAs isomers.

## Methods

### Ethical consideration

The study was performed in agreement with the Declaration of Helsinki principles. Ethical approval was obtained from the Research Ethics Committee of the Federal Medical Center, Keffi Nasarawa State before commencing the study. An informed consent was obtained from the participants or their guardians (if the participant was underage) before enrolment in the study.

### Study design

A cross-sectional analytical study was conducted in adolescents and young adults that volunteered to participate in the study. The volunteers were selected by a non-randomized method following advertisement of the study in the university and senior secondary schools. Patients receiving treatment for acne vulgaris, those that suspended topical or systemic treatments earlier than two weeks (four weeks for patients that used retinoids) and patients with other skin or systemic diseases that could interfere with sebum analysis were excluded from the study. Enrolment was concluded when sixty participants (30 acne patients and 30 controls) that met the requirements were recruited. Equal number of females (F) and males (M) were recruited in both groups with age ranging between 15 and 25 years. The face of acne patients was examined. The types of acne lesions on the face, i.e. non inflammatory (comedones), and inflammatory ones (papules, pustules and nodules), were counted and documented. The severity of acne was rated as mild (1, predominance of comedones and a few inflammatory lesions), moderate (2, some comedones, and mainly papules, and pustules), and severe (3, predominance of papules, pustules and nodules).

### Materials, chemicals, and reagents

Sebutapes and storage cards were purchased from CuDerm Corporation (Dallas, TX, US). Authentic lipid standards were purchased from several manufacturers as detailed in Supplementary Table S2. Other details of the lipid standards are provided in the table. The chemicals to prepare the solution of BSTFA in pyridine containing 1% trimethylchlorosilane (TCMS) were from Merck (Darmstadt, Germany).

### Sebum sampling

Sebum was collected with the Sebutapes adhesive patches. Two patches were placed onto the centre of the forehead and two patches were applied onto the most prominent area of the cheeks, one on each side, to absorb sebum for 30 min. The patches were removed and placed on storage cards, which were labelled appropriately as non-acne controls (NA1–NA30) and acne cases (A1–A30). The cards were stored refrigerated until processing.

### Sample preparation and sebum analysis

Sebum analysis was carried out separately on specimens obtained from the foreheads and the cheeks. To perform the extraction of the study samples and blank tapes, clean glass-tubes were pre-loaded with 200 µL of the internal standard (ISTD) mixture (d6cholesterol 100 µM, d6squalene 100 µM, and d17C16:0 50 µM in ethanol) and stored at − 20 °C until use. Sebum lipids were extracted from the tapes as previously reported^20^. Details are provided in Supplementary Appendix. The dry extract was dissolved in 500 µL of acetone/methanol/isopropanol (40/40/20 v/v/v) mixture to be analyzed by gas chromatography coupled to mass spectrometry (GCMS) and high-performance thin layer chromatography (HPTLC) to determine the target lipid species as detailed in Supplementary Appendix. For the quantitative analyses of individual lipids by GCMS we constructed calibration curves of the authentic standards listed in the Supplementary Table S2, representing FFAs, FOHs, squalene, cholesterol, and vitamin E in sebum. The molecular ions and the fragments accounted for the quantitative GCMS assessments are summarized in the Supplementary Table S2. Amounts of putative known lipids, i.e. FOH C20:0, FOH C22:0, and FOH C24:0, were referred to the structurally closest compound available as an authentic standard. Total TGs, WEs, and cholesterol esters (CEs) were assessed quantitatively by HPTLC, against reference lipids loaded at different concentration on the same plate together with the sebum samples. Representative HPTLC and GCMS chromatograms are reported in S﻿upplementary Fig. S1.

Data were analysed using the statistical and data analysis solutions XLSTAT 2020.1.2 (Addinsoft, New York, USA), and MatLab (version 8.6.0 release R2015b; The Mathworks, Natick, MA). Continuous variables were represented as median values with confidence intervals or mean ± standard deviation (SD). Mann–Whitney and Kruskal–Wallis tests were used for comparison between two or more groups. Spearman’s coefficient (R) was used to measure the correlation between two quantitative variables. Differences and correlations were considered statistically significant with p ≤ 0.05. ANOVA-simultaneous component analysis (ASCA)^44^, was used to determine the effect of the controlled factors in the experimental design on the multivariate chromatographic profiles, as described in Supplementary Appendix.

## ﻿Supplementary Information

Supplementary Information.

Supplementary Figure S1.

Supplementary Figure S2.

Supplementary Figure S3.

Supplementary Table S1.

Supplementary Table S2.

Supplementary Table S3.

Supplementary Legends.
