# Author Correction: Lipidomics of facial sebum in the comparison between acne and non-acne adolescents with dark skin

**DOI:** 10.1038/s41598-021-97802-6

**Published:** 2021-09-03

**Authors:** Obumneme Emeka Okoro, Adebomi Adenle, Matteo Ludovici, Mauro Truglio, Federico Marini, Emanuela Camera

**Affiliations:** 1 Dermatology Unit, Federal Medical Centre, Keffi, Nasarawa, Nigeria; 2 Laboratory of Cutaneous Physiopathology, San Gallicano Dermatological Institute-IRCCS, Rome, Italy; 3 Department of Chemistry, University of Rome ‘La Sapienza’, Rome, Italy

Correction to: *Scientific Reports* 10.1038/s41598-021-96043-x, published online 16 August 2021

The Supplementary Information file published with this Article contained an error in the vertical axis label in Figure S1(a), where the order of labels on the left side was incorrect.

This error has now been corrected in the Supplementary Information file that accompanies the original Article.

