# Supplementary material for: Lipidomics of facial sebum in the comparison between acne and non-acne adolescents with dark skin
Source: Sci Rep. 2021 Aug 16;11:16591. doi: 10.1038/s41598-021-96043-x (PMC8367971; doi:10.1038/s41598-021-96043-x)
Supplement: Supplementary file 1 — Supplementary Information. [file 41598_2021_96043_MOESM1_ESM.docx]

Appendix

**GCMS**

Gas chromatography coupled to mass spectrometry (GCMS) following electron impact ionization was employed to determine quantitatively target compounds in the sebum extracts. Samples were analyzed with a GC 7890A coupled to the MS 5975 VL analyzer (Agilent Technologies, CA, USA). Quantitative analysis of free fatty acids (FFAs), fatty alcohols (FOHs), squalene, cholesterol, vitamin E, and WE(14:0/16:0) was performed by a GCMS method as previously reported ^1-3^. Briefly, 10 µL of the sebum extract dissolved in 50 µL of acetone/methanol/isopropanol mixture (40/40/20 v/v/v) were dried under nitrogen and derivatized with 50 µL BSTFA added of 1% trimethylchlorosilane (TCMS) in pyridine. To generate the trimethylsilyl (TMS) derivatives of FFAs, FOHs, cholesterol, and vitamin E the reaction was carried out at 60 °C for 60 minutes ^4^. GC separation was performed with the 30 m–0.250 (i.d.) GC DB-5MS UI fused silica column (Agilent Technologies, CA, USA), chemically bonded with a 5% diphenyl 95% dimethylpolysiloxane cross-linked stationary phase (0.25 mm film thickness). Helium was used as the carrier gas. Samples were acquired by monitoring the molecular and the fragment ions in scan mode by means of electron impact (EI) MS. The molecular ions and the fragments accounted for the quantitative assessments are summarized in the Supplementary Table S2. A representative GCMS chromatogram is reported in Supplementary Figure S1.

**HPTLC**

Lipid extracts were separated onto silica gel 60 high-performance high performance thin layer chromatography (HPTLC) plates (20x10 cm^2^; Merck, Darmstadt, Germany) ^5^. Ten µL of the dissolved lipid extract from each sample were loaded onto the HPTLC plate. The major sebum lipids (TGs, FFAs, WEs, CEs, cholesterol, and squalene) were separated using benzene/hexane/acetic acid 70/30/0.1 (v/v/v) as the mobile phase in a chromatographic chamber (CAMAG, Muttenz, Switzerland). Quantification was performed against known amounts of authentic cholesterol, FA C16:1, triolein (TG), palmitin palmitoleate (WE), cholesterol oleate (CE), and squalene. Densitometry of the developed spots was performed to assess spot intensities using the ImageJ software package (<http://imagej.nih.gov/ij/>). The optical density of the TGs, WEs, and CEs bands was measured and quantified against the corresponding bands in the standard mixture. To control performance of the HPTLC analysis, pooled sebum samples (quality control, QC) and blank tapes extract were loaded on the same plate for each series of study samples. Extracts from foreheads and cheeks of the same donor were spotted sequentially. Each plate was loaded, in the order, with one blank extract (i.e. mock extraction), four calibration levels of the authentic lipid mixture corresponding to 2, 4, 6, and 8 µg of each component, one QC, sebum extracts from foreheads and cheeks of 3 NA and 3 A donors, and, at the end, extract from blank tapes (Supplementary Figure S1).

**ANOVA-simultaneous component analysis (ASCA)**

Due to the advantage of ASCA of coupling multivariate ANOVA decomposition of the experimental data matrix with non-parametric testing and interpretation of the multivariate effects through the use of principal component analysis (PCA) it was applied to the data matrices on sebum. In detail, by indicating as **X** the matrix collecting the results of the designed experiments, the first step of the procedure is to partition the variability in it, according to the ANOVA scheme, i.e., as a sum of additive terms, each accounting for the effect of a particular design term (factor or interaction). In the case where a single factor is of interest, such as in the present study, the data matrix is decomposed as follows:

**X**=**X**_m_+**X**_acne_+**X**_res_

All these matrices have the same dimensions, but are constructed in different ways. In **X**_m_, each row contains the mean experimental profile calculated on all the samples; indeed, this “grand mean” matrix is introduced just to express the variation induced by the factor(s) as differences with respect to the mean profile. **X**_acne_ is the matrix accounting for the effect of acne; since the factor has only two levels, A and NA, the matrix is built accordingly: All the rows of **X**_acne_ corresponding to samples of patients with acne will contain identical copies of the mean profile recorded on these patients, and the same occurs for the non-acne patients. Lastly, **X**_res_ is the residual matrix, which contains the variability not explained by the linear ANOVA model. Significance of the effect can be estimated by the sum of squares (SSQ) of the elements of the effect matrix **X**_acne_ and its statistical significance may be evaluated by comparing the experimental SSQ with its distribution under the null hypothesis, which can be obtained by permutation tests ^6^. If the particular design term is found to have a significant effect, then interpretation can be carried out by calculating a principal component analysis model of the effect matrix (in the present case, **X**_acne_), which allows evaluating what are the changes in the multivariate experimental profile induced by the different levels of the controlled factor(s).

**Supplementary references**

1 Torocsik D, Kovacs D, Camera E, et al. Leptin promotes a proinflammatory lipid profile and induces inflammatory pathways in human SZ95 sebocytes. *Br J Dermatol* 2014; **171**:1326-35.

2 Argus JP, Yu AK, Wang ES, et al. An optimized method for measuring fatty acids and cholesterol in stable isotope-labeled cells. *J Lipid Res* 2017; **58**:460-8.

3 Singh K, Camera E, Krug L, et al. JunB defines functional and structural integrity of the epidermo-pilosebaceous unit in the skin. *Nat Commun* 2018; **9**:3425,018-05726-z.

4 Pasikanti KK, Ho PC, Chan EC. Gas chromatography/mass spectrometry in metabolic profiling of biological fluids. *J Chromatogr B Analyt Technol Biomed Life Sci* 2008; **871**:202-11.

5 Bernard-Savary P, Poole CF. Instrument platforms for thin-layer chromatography. *J Chromatogr A* 2015; **1421**:184-202.

6 Vis DJ, Westerhuis JA, Smilde AK, van der Greef J. Statistical validation of megavariate effects in ASCA. *BMC Bioinformatics* 2007; **8**:322,2105-8-322.
