# Supplementary material for: Lipidomics of facial sebum in the comparison between acne and non-acne adolescents with dark skin
Source: Sci Rep. 2021 Aug 16;11:16591. doi: 10.1038/s41598-021-96043-x (PMC8367971; doi:10.1038/s41598-021-96043-x)
Supplement: Supplementary file 2 — Supplementary Figure S1. [file 41598_2021_96043_MOESM2_ESM.pptx]

## Slide 1
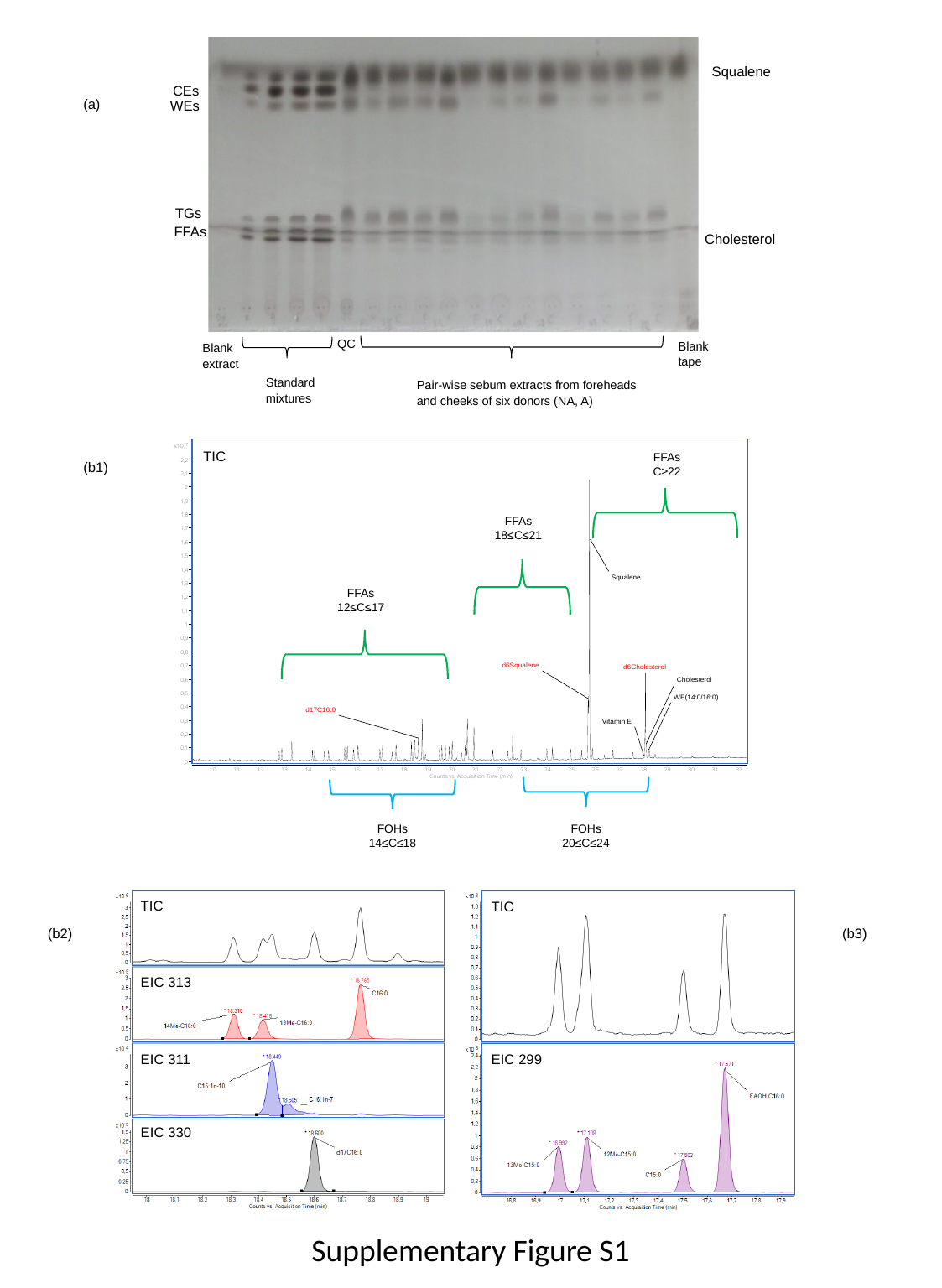

Squalene
CEs
(a)
WEs
TGs
FFAs
Cholesterol
QC
Blank tape
Blank extract
Standard mixtures
Pair-wise sebum extracts from foreheads and cheeks of six donors (NA, A)
TIC
FFAs
C≥22
(b1)
FFAs
18≤C≤21
FFAs
12≤C≤17
FOHs
20≤C≤24
FOHs
14≤C≤18
TIC
TIC
(b2)
(b3)
EIC 313
EIC 299
EIC 311
EIC 330
Supplementary Figure S1
