# Supplementary material for: Lipidomics of facial sebum in the comparison between acne and non-acne adolescents with dark skin
Source: Sci Rep. 2021 Aug 16;11:16591. doi: 10.1038/s41598-021-96043-x (PMC8367971; doi:10.1038/s41598-021-96043-x)
Supplement: Supplementary file 3 — Supplementary Figure S2. [file 41598_2021_96043_MOESM3_ESM.pptx]

## Slide 1
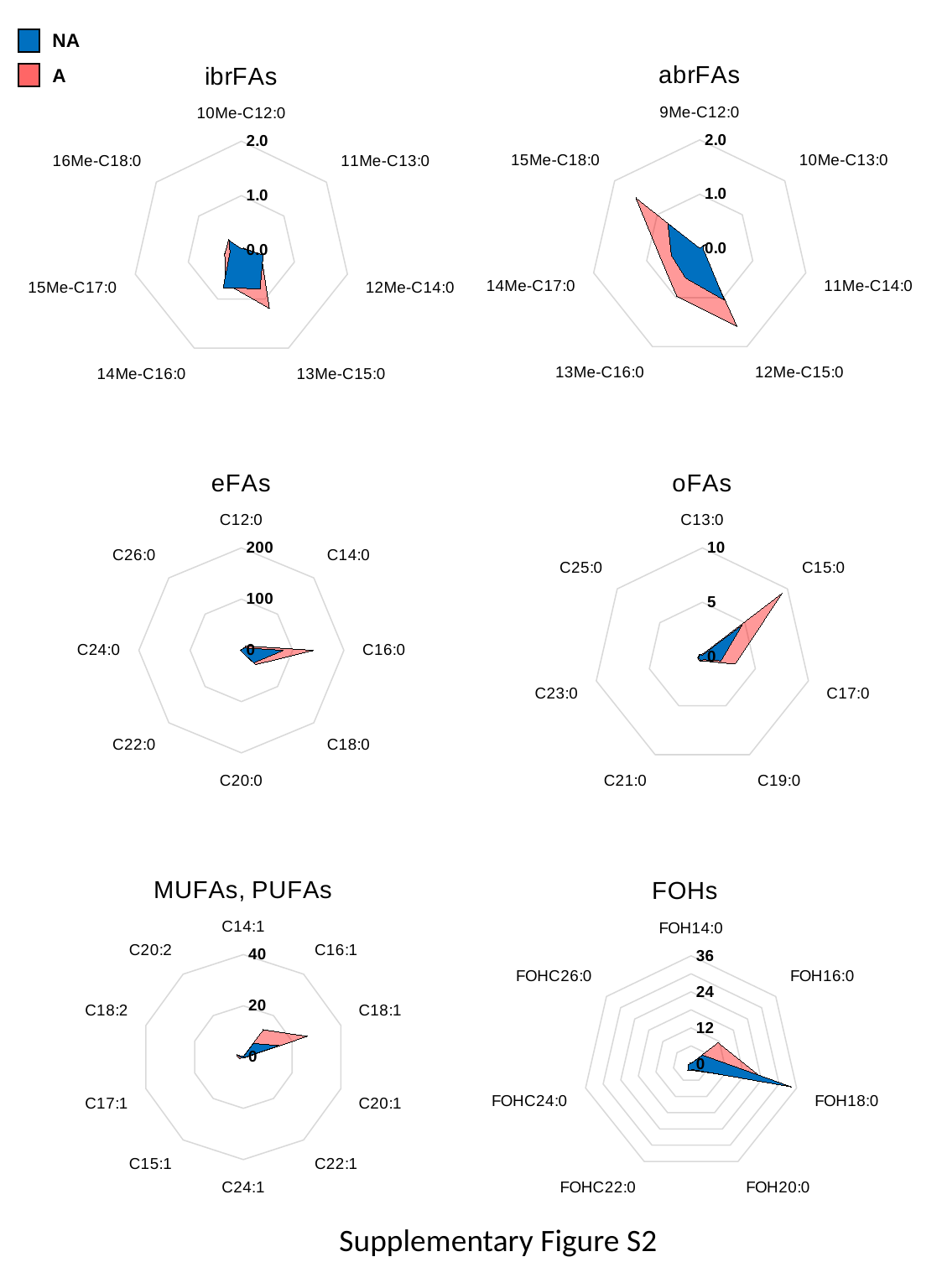

NA
### Chart: abrFAs
| Category | NA | A |
|---|---|---|
| 9Me-C12:0 | 0.011795899699250649 | 0.007083570652465973 |
| 10Me-C13:0 | 0.09056970172911011 | 0.12623129719924517 |
| 11Me-C14:0 | 0.07359185838307684 | 0.04183795227731994 |
| 12Me-C15:0 | 1.051651321083449 | 1.586696116259031 |
| 13Me-C16:0 | 0.5967026293367751 | 0.9691961400456912 |
| 14Me-C17:0 | 0.5339082106910067 | 0.7393928581748216 |
| 15Me-C18:0 | 0.7487841776672138 | 1.5008719416127885 |
### Chart: ibrFAs
| Category | NA | A |
|---|---|---|
| 10Me-C12:0 | 0.026966046360399905 | 0.02229785304723377 |
| 11Me-C13:0 | 0.04250792333931064 | 0.057753748047575236 |
| 12Me-C14:0 | 0.40954231278387465 | 0.372654712268532 |
| 13Me-C15:0 | 0.7919635100589663 | 1.1909994663227355 |
| 14Me-C16:0 | 0.7715068374821751 | 0.6757447521299533 |
| 15Me-C17:0 | 0.2174039989215571 | 0.31328713118399115 |
| 16Me-C18:0 | 0.27290079074166224 | 0.30178076923419533 |A
### Chart: eFAs
| Category | NA | A |
|---|---|---|
| C12:0 | 1.0784122530465583 | 1.1353615226181875 |
| C14:0 | 6.673495771854634 | 12.081352542833322 |
| C16:0 | 81.93735940429718 | 140.83770424152308 |
| C18:0 | 34.06816179666274 | 39.27426631309865 |
| C20:0 | 0.7558386668717793 | 1.1039388829552501 |
| C22:0 | 0.8268470668521289 | 1.0938769171022948 |
| C24:0 | 1.0754617896057979 | 1.5774341951022077 |
| C26:0 | 0.15530534910317698 | 0.23540188786981062 |
### Chart: oFAs
| Category | NA | A |
|---|---|---|
| C13:0 | 0.1565059407945037 | 0.24099889723676723 |
| C15:0 | 4.651959633108802 | 9.351453769646083 |
| C17:0 | 1.7415952492879876 | 3.080857017989412 |
| C19:0 | 0.31993615456423835 | 0.44783323619886667 |
| C21:0 | 0.37653818720806476 | 0.44824829073627803 |
| C23:0 | 0.3618151081465228 | 0.45970926952393104 |
| C25:0 | 0.30067348482171163 | 0.35041125286878955 |
### Chart: MUFAs, PUFAs
| Category | NA | A |
|---|---|---|
| C14:1 | 0.2649187912116189 | 0.4322340455686355 |
| C16:1 | 6.554454588316273 | 13.204227997118247 |
| C18:1 | 14.470479646109867 | 26.10932138924754 |
| C20:1 | 0.4080486377642378 | 0.5975477879291727 |
| C22:1 | 0.07035661426510285 | 0.09604795238056676 |
| C24:1 | 0.11172727474979344 | 0.16196744822230874 |
| C15:1 | 0.22043555113466815 | 0.4229730867074479 |
| C17:1 | 0.8541156390475443 | 1.7070858575868009 |
| C18:2 | 2.1768251160141863 | 2.7248940075056267 |
| C20:2 | 0.3451135407927478 | 0.3691995763930426 |
### Chart: FOHs
| Category | NA | A |
|---|---|---|
| FOH14:0 | 0.2075110551729783 | 0.2570437463018451 |
| FOH16:0 | 4.81170056917695 | 11.491807873219516 |
| FOH18:0 | 34.292882355218985 | 25.732211633088934 |
| FOH20:0 | 2.162745064376367 | 2.4415189724267297 |
| FOHC22:0 | 2.2925068678307357 | 2.512875640715163 |
| FOHC24:0 | 0.857027795717526 | 0.9808381996067557 |
| FOHC26:0 | 0.4056225872158769 | 0.4448698991189971 |Supplementary Figure S2
