# Supplementary material for: Lipidomics of facial sebum in the comparison between acne and non-acne adolescents with dark skin
Source: Sci Rep. 2021 Aug 16;11:16591. doi: 10.1038/s41598-021-96043-x (PMC8367971; doi:10.1038/s41598-021-96043-x)
Supplement: Supplementary file 4 — Supplementary Figure S3. [file 41598_2021_96043_MOESM4_ESM.pptx]

## Slide 1
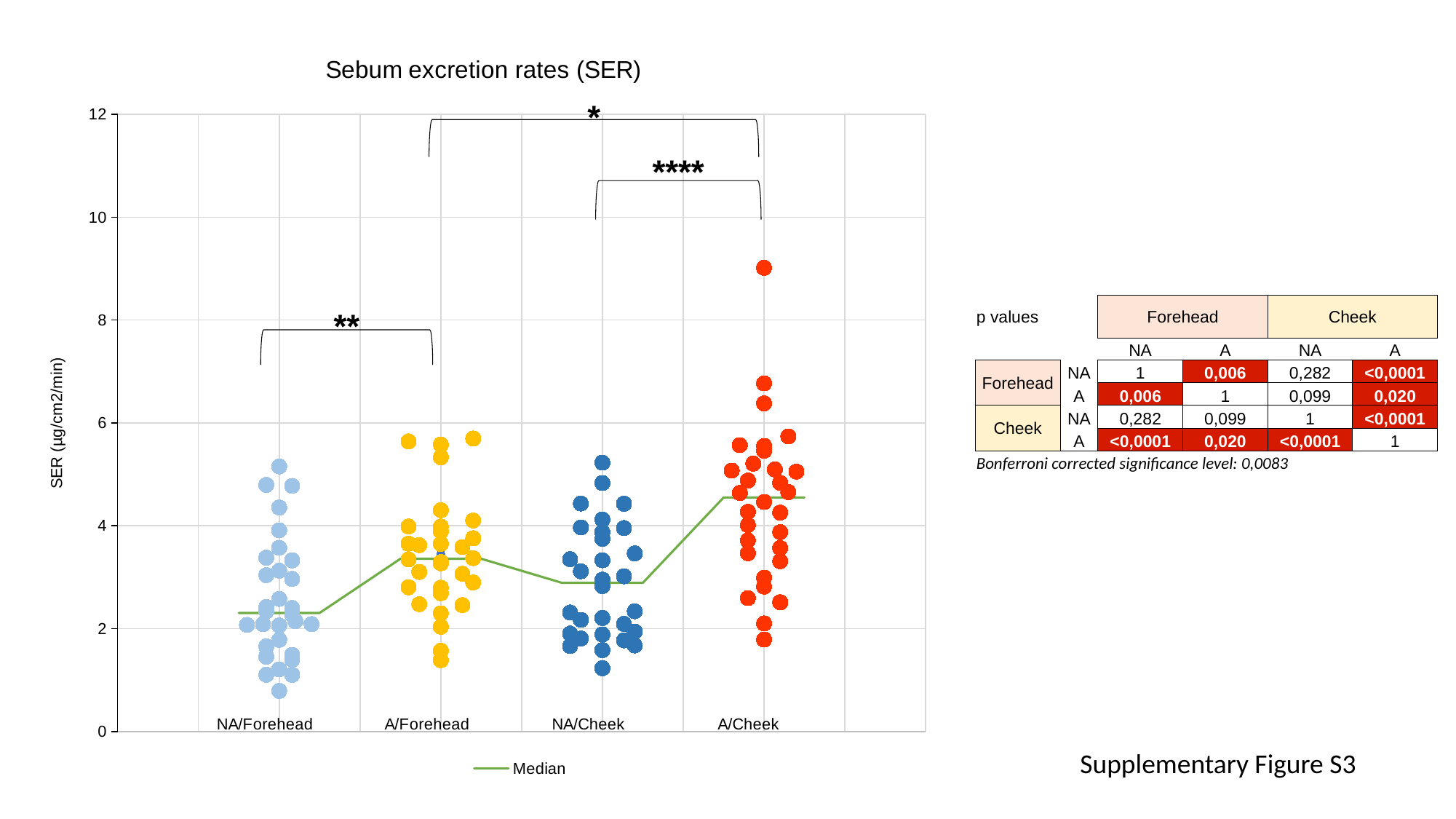

### Chart: Sebum excretion rates (SER)
| Category | | | | | | | |
|---|---|---|---|---|---|---|---|
*
****
**
| p values | | Forehead | | Cheek | |
| --- | --- | --- | --- | --- | --- |
| | | NA | A | NA | A |
| Forehead | NA | 1 | 0,006 | 0,282 | <0,0001 |
| | A | 0,006 | 1 | 0,099 | 0,020 |
| Cheek | NA | 0,282 | 0,099 | 1 | <0,0001 |
| | A | <0,0001 | 0,020 | <0,0001 | 1 |
| Bonferroni corrected significance level: 0,0083 | | | | | |
Supplementary Figure S3
