# Supplementary material for: Lipidomics of facial sebum in the comparison between acne and non-acne adolescents with dark skin
Source: Sci Rep. 2021 Aug 16;11:16591. doi: 10.1038/s41598-021-96043-x (PMC8367971; doi:10.1038/s41598-021-96043-x)
Supplement: Supplementary file 5 — Supplementary Table S1. [file 41598_2021_96043_MOESM5_ESM.docx]

Supplementary Table S1. Demographics and acne severity.

| **ID** | AGE | GENDER |  | **ID** | AGE | GENDER | COMEDONES | PAPULES | PUSTULES | NODULES | TOTAL LESION COUNT | SEVERITY |
| --- | --- | --- | --- | --- | --- | --- | --- | --- | --- | --- | --- | --- |
| **NA1** | 19 | M |  | **A1** | 20 | F | 18 | 7 | 0 | 0 | 25 | 1 |
| **NA2** | 21 | M |  | **A2** | 17 | F | 13 | 10 | 0 | 0 | 23 | 1 |
| **NA3** | 21 | M |  | **A3** | 17 | M | 17 | 12 | 0 | 0 | 29 | 1 |
| **NA4** | 16 | F |  | **A4** | 17 | M | 15 | 8 | 0 | 0 | 23 | 1 |
| **NA5** | 16 | F |  | **A5** | 17 | M | 19 | 34 | 2 | 0 | 55 | 2 |
| **NA6** | 15 | F |  | **A6** | 19 | M | 10 | 18 | 0 | 0 | 28 | 1 |
| **NA7** | 17 | M |  | **A7** | 16 | F | 32 | 49 | 6 | 0 | 87 | 3 |
| **NA8** | 15 | F |  | **A8** | 18 | M | 31 | 21 | 0 | 0 | 52 | 2 |
| **NA9** | 15 | F |  | **A9** | 19 | M | 38 | 41 | 3 | 0 | 82 | 3 |
| **NA10** | 16 | M |  | **A10** | 15 | M | 9 | 11 | 0 | 0 | 20 | 1 |
| **NA11** | 19 | M |  | **A11** | 15 | F | 28 | 19 | 1 | 0 | 48 | 2 |
| **NA12** | 18 | M |  | **A12** | 18 | F | 18 | 11 | 0 | 1 | 30 | 2 |
| **NA13** | 19 | M |  | **A13** | 18 | F | 15 | 9 | 0 | 0 | 24 | 1 |
| **NA14** | 16 | M |  | **A14** | 16 | F | 30 | 18 | 1 | 0 | 49 | 2 |
| **NA15** | 17 | F |  | **A15** | 16 | F | 43 | 28 | 3 | 1 | 75 | 3 |
| **NA16** | 20 | F |  | **A16** | 17 | F | 39 | 17 | 0 | 0 | 56 | 2 |
| **NA17** | 17 | F |  | **A17** | 15 | F | 21 | 8 | 1 | 0 | 30 | 1 |
| **NA18** | 19 | F |  | **A18** | 16 | M | 18 | 33 | 5 | 0 | 56 | 2 |
| **NA19** | 18 | F |  | **A19** | 15 | F | 19 | 5 | 0 | 0 | 24 | 1 |
| **NA20** | 17 | F |  | **A20** | 17 | M | 18 | 7 | 0 | 0 | 25 | 1 |
| **NA21** | 16 | F |  | **A21** | 17 | M | 13 | 17 | 0 | 0 | 30 | 1 |
| **NA22** | 18 | F |  | **A22** | 16 | M | 41 | 52 | 4 | 0 | 97 | 3 |
| **NA23** | 18 | F |  | **A23** | 17 | M | 20 | 34 | 2 | 0 | 56 | 2 |
| **NA24** | 15 | F |  | **A24** | 15 | F | 22 | 14 | 3 | 0 | 39 | 2 |
| **NA25** | 17 | M |  | **A25** | 16 | F | 19 | 21 | 0 | 0 | 40 | 2 |
| **NA26** | 19 | M |  | **A26** | 20 | M | 19 | 21 | 8 | 2 | 50 | 3 |
| **NA27** | 20 | M |  | **A27** | 21 | M | 13 | 18 | 0 | 0 | 31 | 1 |
| **NA28** | 18 | M |  | **A28** | 19 | F | 21 | 18 | 0 | 0 | 39 | 2 |
| **NA29** | 16 | M |  | **A29** | 17 | F | 15 | 9 | 0 | 0 | 24 | 1 |
| **NA30** | 18 | M |  | **A30** | 21 | M | 12 | 19 | 0 | 0 | 31 | 1 |
