# Supplementary material for: Lipidomics of facial sebum in the comparison between acne and non-acne adolescents with dark skin
Source: Sci Rep. 2021 Aug 16;11:16591. doi: 10.1038/s41598-021-96043-x (PMC8367971; doi:10.1038/s41598-021-96043-x)
Supplement: Supplementary file 6 — Supplementary Table S2. [file 41598_2021_96043_MOESM6_ESM.docx]

Supplementary Table S2. List of lipid metabolites determined in sebum by GCMS.

| **Systematic name** | **Supplier** | **LIPIDMAPS ID** | **Common name** | **Synonyms** | **FORMULAE** | **MW** | **[M+TMS]** | **[M+TMS-15]** | **RT (average)** | **Type** | **Label** |
| --- | --- | --- | --- | --- | --- | --- | --- | --- | --- | --- | --- |
| 10-Methylundecanoic acid | LARODAN | LMFA01020004 | Isolauric acid | 10Me-C12:0 | C12H24O2 | 200,18 | 272,18 | **257,18** | 12,8 | isobranched | ibrFA |
| 11-Methyldodecanoic acid | LARODAN | LMFA01020006 | 11-Methyl lauric acid | 11Me-C13:0 | C13H26O2 | 214,19 | 286,19 | **271,19** | 14,2 | isobranched | ibrFA |
| 12-Methyltridecanoic acid | LARODAN | LMFA01020007 | (+)-Isomyristic acid | 12Me-C14:0 | C14H28O2 | 228,21 | 300,21 | **285,21** | 15,6 | isobranched | ibrFA |
| 13-Methyltetradecanoic acid | LARODAN | LMFA01020009 | Isopentadecylic acid | 13Me-C15:0 | C15H30O2 | 242,22 | 314,22 | **299,22** | 17,0 | isobranched | ibrFA |
| 14-Methylpentadecanoic acid | LARODAN | LMFA01020010 | Isopalmitic acid | 14Me-C16:0 | C16H32O2 | 256,24 | 328,24 | **313,24** | 18,3 | isobranched | ibrFA |
| 15-Methylhexadecanoic acid | LARODAN | LMFA01020012 | Isomargaric acid | 15Me-C17:0 | C17H34O2 | 270,26 | 342,26 | **327,26** | 19,5 | isobranched | ibrFA |
| 16-Methylheptadecanoic acid | LARODAN | LMFA01020014 | Isooctadecanoic acid | 16Me-C18:0 | C18H36O2 | 284,27 | 356,27 | **341,27** | 20,6 | isobranched | ibrFA |
| 9-Methylundecanoic acid | LARODAN | LMFA01020059 |  | 9Me-C12:0 | C12H24O2 | 200,18 | 272,18 | **257,18** | 13,0 | anteisobranched | abrFA |
| 10-Methyldodecanoic acid | LARODAN | LMFA01020005 |  | 10Me-C13:0 | C13H26O2 | 214,19 | 286,19 | **271,19** | 14,3 | anteisobranched | abrFA |
| 11-Methyltridecanoic acid | LARODAN | LMFA01020190 |  | 11Me-C14:0 | C14H28O2 | 228,21 | 300,21 | **285,21** | 15,7 | anteisobranched | abrFA |
| 12-Methyltetradecanoic acid | LARODAN | LMFA01020008 |  | 12Me-C15:0 | C15H30O2 | 242,22 | 314,22 | **299,22** | 17,1 | anteisobranched | abrFA |
| 13-Methylpentadecanoic acid | LARODAN | LMFA01020192 |  | 13Me-C16:0 | C16H32O2 | 256,24 | 328,24 | **313,24** | 18,4 | anteisobranched | abrFA |
| 14-Methylhexadecanoic acid | LARODAN | LMFA01020011 | Anteisomargaric acid | 14Me-C17:0 | C17H34O2 | 270,26 | 342,26 | **327,26** | 19,6 | anteisobranched | abrFA |
| 15-Methylheptadecanoic acid | LARODAN | LMFA01020205 |  | 15Me-C18:0 | C18H36O2 | 284,27 | 356,27 | **341,27** | 20,7 | anteisobranched | abrFA |
| Dodecanoic acid | LARODAN | LMFA01010012 | Lauric acid | C12:0 | C12H24O2 | 200,18 | 272,18 | **257,18** | 13,4 | even | eFA |
| Tetradecanoic acid | LARODAN | LMFA01010014 | Myristic acid | C14:0 | C14H28O2 | 228,21 | 300,21 | **285,21** | 16,1 | even | eFA |
| Hexadecanoic acid | LARODAN | LMFA01010001 | Palmitic acid | C16:0 | C16H32O2 | 256,24 | 328,24 | **313,24** | 18,8 | even | eFA |
| Octadecanoic acid | LARODAN | LMFA01010018 | Stearic acid | C18:0 | C18H36O2 | 284,27 | 356,27 | **341,27** | 21,0 | even | eFA |
| Eicosanoic acid | LARODAN | LMFA01010020 | Arachidic acid | C20:0 | C20H40O2 | 312,30 | 384,30 | **369,30** | 23,8 | even | eFA |
| Docosanoic acid | LARODAN | LMFA01010022 | Behenic acid | C22:0 | C22H44O2 | 340,33 | 412,33 | **397,33** | 26,3 | even | eFA |
| Tetracosanoic acid | LARODAN | LMFA01010024 | Lignoceric acid | C24:0 | C24H48O2 | 368,36 | 440,36 | **425,36** | 28,9 | even | eFA |
| Hexacosanoic acid | LARODAN | LMFA01010026 | Cerotic acid | C26:0 | C26H52O2 | 396,39 | 468,39 | **453,39** | 31,5 | even | eFA |
| Tridecanoic acid | LARODAN | LMFA01010013 |  | C13:0 | C13H26O2 | 214,19 | 286,19 | **271,19** | 14,7 | odd | oFA |
| Pentadecanoic acid | LARODAN | LMFA01010015 |  | C15:0 | C15H30O2 | 242,22 | 314,22 | **299,22** | 17,5 | odd | oFA |
| Heptadecanoic acid | LARODAN | LMFA01010017 | Margarinic acid | C17:0 | C17H34O2 | 270,26 | 342,26 | **327,26** | 20,0 | odd | oFA |
| Nonadecanoic acid | LARODAN | LMFA01010019 |  | C19:0 | C19H38O2 | 298,29 | 370,29 | **355,29** | 22,5 | odd | oFA |
| Heneicosanoic acid | LARODAN | LMFA01010021 |  | C21:0 | C21H42O2 | 326,32 | 398,32 | **383,32** | 25,0 | odd | oFA |
| Tricosanoic acid | LARODAN | LMFA01010023 |  | C23:0 | C23H46O2 | 354,35 | 426,35 | **411,35** | 27,6 | odd | oFA |
| Pentacosanoic acid | LARODAN | LMFA01010025 | Hyenic acid | C25:0 | C25H50O2 | 382,38 | 454,38 | **439,38** | 30,2 | odd | oFA |
| 9Z-Tetradecenoic acid | LARODAN | LMFA01030051 |  | C14:1 | C14H26O2 | 226,19 | 298,19 | **283,19** | 15,7 | monounsaturated | MUFA |
| 6Z-Hexadecenoic acid | LARODAN | LMFA01030267 | Sapienic acid | C16:1n-10 | C16H30O2 | 254,22 | 326,22 | **311,22** | 18,4 | monounsaturated | MUFA |
| 9Z-Hexadecenoic acid | LARODAN | LMFA01030056 | cis-9-palmitoleic acid | C16:1n-7 | C16H30O2 | 254,22 | 326,22 | **311,22** | 18,9 | monounsaturated | MUFA |
| 9Z-octadecenoic acid | LARODAN | LMFA01030002 |  | C18:1 | C18H34O2 | 282,26 | 354,26 | **339,26** | 20,8 | monounsaturated | MUFA |
| 10Z-Eicosenoic acid | Tentatively assigned | LMFA01031093 |  | C20:1 | C20H38O2 | 310,29 | 382,29 | **367,29** | 21,9 | monounsaturated | MUFA |
| 9Z,12Z-Octadecadienoic acid | LARODAN | LMFA01030120 |  | C18:2 | C18H32O2 | 280,24 | 352,24 | **337,24** | 20,6 | polyunsaturated | PUFA |
| 11,14-Eicosadienoic acid | Cayman Chemical | LMFA01030130 |  | C20:2 | C20H36O2 | 308,27 | 380,27 | **365,27** | 22,5 | polyunsaturated | PUFA |
| 1-Tetradecanol | TRC | LMFA05000041 |  | FAOH C14:0 | C14H30O | 214,23 | 286,23 | **271,23** | 14,9 | Fatty alcohol | FOH |
| 1-Hexadecanol | TRC | LMFA05000061 | Cetyl alcohol | FAOH C16:0 | C16H34O | 242,26 | 314,26 | **299,26** | 17,7 | Fatty alcohol | FOH |
| Octadecan-1-ol | TRC | LMFA05000085 | Stearyl alcohol | FAOH C18:0 | C18H38O | 270,29 | 342,29 | **327,29** | 20,1 | Fatty alcohol | FOH |
| Eicosan-1-ol | Tentatively assigned | LMFA05000221 |  | FAOH C20:0 | C20H42O | 298,32 | 370,32 | **355,32** | 22,7 | Fatty alcohol | FOH |
| 1-docosanol | Tentatively assigned | LMFA05000008 | Behenyl alcohol | FAOH C22:0 | C22H46O | 326,35 | 398,35 | **383,35** | 25,3 | Fatty alcohol | FOH |
| Tetracosan-1-ol | Tentatively assigned | LMFA05000222 | Lignoceryl alcohol | FAOH C24:0 | C24H50O | 354,39 | 426,39 | **411,39** | 27,9 | Fatty alcohol | FOH |
| α-tocopherol | SIGMA-ALDRICH | LMPR02020001 | Vitamin E | Vitamin E | C29H50O2 | 430,38 | **502,38** | 487,38 | 28,0 |  |  |
| Cholest-5-en-3β-ol | TRC | LMST01010001 | Cholesterol | Cholesterol | C27H46O | 386,35 | **458,35** | 443,35 | 28,1 |  |  |
| Squalene | TRC | LMPR0106010002 | Squalene | Squalene | C30H50 | **410,39** | NA | NA | 25,8 |  |  |
| d17Palmitate | C/D/N Isotopes | Deuterated ISTD | d17Palmitate | d17C16:0 | C16H15D17O2 | 273,53 | 345,53 | **330,53** | 18,6 |  |  |
| d6Squalene | TRC | Deuterated ISTD | d6Squalene | d6Squalene | C30H44D6 | **416,75** | NA | NA | 25,7 |  |  |
| d6Cholesterol | C/D/N Isotopes | Deuterated ISTD | d6Cholesterol | d6Cholesterol | C27H40D6O | 392,70 | **464,70** | 449,70 | 28,1 |  |  |

Legend

TMS= Trimethyl silyl derivative

NA= Not applicable

The m/z ratio in bold were accounted for the quantitative assessments
