# Supplementary material for: Lipidomics of facial sebum in the comparison between acne and non-acne adolescents with dark skin
Source: Sci Rep. 2021 Aug 16;11:16591. doi: 10.1038/s41598-021-96043-x (PMC8367971; doi:10.1038/s41598-021-96043-x)
Supplement: Supplementary file 7 — Supplementary Table S3. [file 41598_2021_96043_MOESM7_ESM.docx]

**Supplementary Table S3.** Average amounts (µg) and standard deviation (SD) of sebum lipids in non acne (NA) and acne (A) groups.

| **Forehead (µg)** | **NA** | **A** | SD NA | SD A | FC | Significance |
| --- | --- | --- | --- | --- | --- | --- |
| 10Me-C12:0 | 0,024 | 0,019 | 0,025 | 0,0094 | 0,765 | ns |
| 11Me-C13:0 | 0,036 | 0,042 | 0,023 | 0,0163 | 1,180 | * |
| 12Me-C14:0 | 0,299 | 0,272 | 0,447 | 0,1470 | 0,909 | ns |
| 13Me-C15:0 | 0,618 | 0,869 | 0,431 | 0,3720 | 1,405 | *** |
| 14Me-C16:0 | 0,577 | 0,483 | 0,923 | 0,2957 | 0,837 | ns |
| 15Me-C17:0 | 0,179 | 0,226 | 0,112 | 0,0881 | 1,264 | * |
| 16Me-C18:0 | 0,390 | 0,459 | 0,787 | 0,6430 | 1,177 | ns |
| ibrFAs | 2,124 | 2,370 | 2,325 | 1,0202 | 1,116 | * |
| 9Me-C12:0 | 0,007 | 0,005 | 0,009 | 0,0080 | 0,685 | ns |
| 10Me-C13:0 | 0,063 | 0,076 | 0,051 | 0,0241 | 1,200 | ** |
| 11Me-C14:0 | 0,025 | 0,018 | 0,019 | 0,0113 | 0,719 | ns |
| 12Me-C15:0 | 0,755 | 1,064 | 0,635 | 0,3948 | 1,409 | **** |
| 13Me-C16:0 | 0,630 | 0,570 | 0,997 | 0,5118 | 0,905 | ns |
| 14Me-C17:0 | 0,411 | 0,509 | 0,341 | 0,1786 | 1,240 | *** |
| 15Me-C18:0 | 0,931 | 1,472 | 0,793 | 0,7387 | 1,582 | **** |
| abrFAs | 2,822 | 3,714 | 2,748 | 1,5754 | 1,316 | **** |

| **Forehead (µg)** | **NA** | **A** | SD NA | SD A | FC | Significance |
| --- | --- | --- | --- | --- | --- | --- |
| C12:0 | 0,949 | 1,075 | 0,634 | 0,6409 | 1,132 | ns |
| C14:0 | 5,973 | 9,422 | 5,058 | 4,4651 | 1,577 | **** |
| C16:0 | 77,7 | 108,4 | 64,688 | 50,6229 | 1,395 | *** |
| C18:0 | 38,83 | 42,75 | 31,296 | 21,8145 | 1,101 | ns |
| C20:0 | 0,439 | 0,541 | 0,835 | 0,7641 | 1,234 | *** |
| C22:0 | 0,759 | 0,932 | 0,485 | 0,3707 | 1,229 | ** |
| C24:0 | 1,078 | 1,385 | 1,126 | 0,8414 | 1,286 | ** |
| C26:0 | 0,147 | 0,194 | 0,241 | 0,1791 | 1,319 | ns |
| eFAs | 125,9 | 164,7 | 93,813 | 67,9716 | 1,308 | **** |
| C13:0 | 0,137 | 0,208 | 0,089 | 0,0762 | 1,523 | **** |
| C15:0 | 3,909 | 7,194 | 3,661 | 4,0004 | 1,840 | **** |
| C17:0 | 1,541 | 2,521 | 1,217 | 1,2435 | 1,635 | **** |
| C19:0 | 0,320 | 0,386 | 0,332 | 0,2005 | 1,206 | * |
| C21:0 | 0,119 | 0,149 | 0,067 | 0,0593 | 1,255 | ** |
| C23:0 | 0,196 | 0,199 | 0,218 | 0,1149 | 1,012 | ns |
| C25:0 | 0,075 | 0,096 | 0,079 | 0,0488 | 1,283 | *** |
| oFAs | 6,298 | 10,753 | 5,358 | 5,5788 | 1,708 | **** |

| **Forehead (µg)** | **NA** | **A** | SD NA | SD A | FC | Significance |
| --- | --- | --- | --- | --- | --- | --- |
| C14:1 | 0,220 | 0,319 | 0,257 | 0,1456 | 1,453 | **** |
| C15:1 | 0,135 | 0,223 | 0,215 | 0,1809 | 1,657 | **** |
| C16:1 | 5,581 | 6,903 | 8,294 | 3,3252 | 1,237 | **** |
| C17:1 | 0,671 | 1,158 | 0,782 | 0,6648 | 1,726 | **** |
| 14Me-C17:1 | 0,099 | 0,143 | 0,111 | 0,0679 | 1,436 | **** |
| 15Me-C17:1 | 0,051 | 0,074 | 0,054 | 0,0333 | 1,445 | **** |
| C18:1 | 12,63 | 19,99 | 11,581 | 9,6081 | 1,582 | **** |
| C20:1 | 0,377 | 0,464 | 0,406 | 0,2125 | 1,230 | * |
| C22:1 | 0,086 | 0,100 | 0,096 | 0,0568 | 1,164 | * |
| C24:1 | 0,112 | 0,153 | 0,111 | 0,0939 | 1,367 | * |
| MUFAs | 19,96 | 29,52 | 21,380 | 12,4682 | 1,479 | **** |
| C18:2 | 1,917 | 2,489 | 0,556 | 0,6436 | 1,299 | **** |
| C20:2 | 0,153 | 0,228 | 0,140 | 0,1806 | 1,490 | * |
| PUFAs | 2,070 | 2,717 | 0,651 | 0,7046 | 1,313 | **** |
| FFAs | 316,31 | 424,90 | 238,827 | 160,4205 | 1,343 | ** |
| FOHC14:0 | 0,195 | 0,249 | 0,068 | 0,0675 | 1,276 | *** |
| FOHC16:0 | 4,884 | 15,972 | 8,146 | 50,6670 | 3,270 | ns |
| FOHC18:0 | 50,33 | 28,24 | 106,251 | 42,8171 | 0,561 | ns |
| FOHC20:0 | 2,072 | 2,264 | 0,752 | 0,5815 | 1,093 | ns |
| FOHC22:0 | 2,267 | 2,392 | 0,702 | 0,5092 | 1,055 | ns |
| FOHC24:0 | 0,788 | 0,894 | 0,263 | 0,1952 | 1,135 | * |
| FOHC26:0 | 0,268 | 0,268 | 0,321 | 0,1423 | 1,001 | ns |
| FOHs | 60,81 | 50,28 | 111,225 | 81,3426 | 0,827 | ns |

| **Forehead (µg)** | **NA** | **A** | SD NA | SD A | FC | Significance |
| --- | --- | --- | --- | --- | --- | --- |
| Vitamin E | 0,850 | 1,030 | 0,460 | 0,4753 | 1,212 | * |
| Cholesterol | 11,67 | 14,75 | 4,616 | 5,8698 | 1,264 | * |
| Squalene | 129,6 | 223,7 | 94,059 | 102,1004 | 1,726 | **** |
| TGs | 227,39 | 334,04 | 116,840 | 133,2682 | 1,469 | *** |
| WEs | 147,68 | 197,14 | 81,114 | 77,3704 | 1,335 | ** |
| CEs | 124,33 | 127,04 | 26,680 | 19,4028 | 1,022 | ns |
| Sebum | 1017,8 | 1371,8 | 485,38 | 429,82 | 1,348 | ** |
| C16:1/C16:0 | 5,875 | 6,722 | 3,287 | 2,4853 | 1,144 | * |
| C17:1/C17:0 | 37,978 | 45,265 | 10,686 | 7,6802 | 1,192 | ** |
| C18:1/C18:0 | 36,777 | 51,783 | 23,828 | 22,5139 | 1,408 | *** |

| **Cheek (µg)** | **NA** | **A** | SD NA | SD A | FC | Significance |
| --- | --- | --- | --- | --- | --- | --- |
| 10Me-C12:0 | 0,030 | 0,026 | 0,036 | 0,023 | 0,8777 | ns |
| 11Me-C13:0 | 0,049 | 0,073 | 0,040 | 0,049 | 1,4882 | * |
| 12Me-C14:0 | 0,520 | 0,473 | 0,585 | 0,385 | 0,9104 | ns |
| 13Me-C15:0 | 0,966 | 1,513 | 0,521 | 1,377 | 1,5672 | ns |
| 14Me-C16:0 | 0,966 | 0,869 | 1,247 | 0,725 | 0,8990 | ns |
| 15Me-C17:0 | 0,256 | 0,400 | 0,207 | 0,274 | 1,5650 | * |
| 16Me-C18:0 | 0,156 | 0,144 | 0,126 | 0,092 | 0,9277 | ns |
| ibrFAs | 2,942 | 3,499 | 2,360 | 2,510 | 1,1894 | ns |
| 9Me-C12:0 | 0,017 | 0,009 | 0,031 | 0,008 | 0,5650 | ns |
| 10Me-C13:0 | 0,118 | 0,177 | 0,090 | 0,111 | 1,4972 | ** |
| 11Me-C14:0 | 0,122 | 0,065 | 0,420 | 0,149 | 0,5372 | ns |
| 12Me-C15:0 | 1,348 | 2,109 | 0,919 | 1,611 | 1,5649 | ** |
| 13Me-C16:0 | 0,563 | 1,368 | 0,635 | 2,929 | 2,4301 | ** |
| 14Me-C17:0 | 0,657 | 0,970 | 0,392 | 0,948 | 1,4754 | ns |
| 15Me-C18:0 | 0,567 | 1,529 | 0,922 | 3,893 | 2,6976 | ns |
| abrFAs | 3,392 | 6,228 | 2,862 | 9,129 | 1,8363 | ** |

| **Cheek (µg)** | **NA** | **A** | SD NA | SD A | FC | Significance |
| --- | --- | --- | --- | --- | --- | --- |
| C12:0 | 1,207 | 1,196 | 0,797 | 0,927 | 0,9907 | ns |
| C14:0 | 7,374 | 14,741 | 4,380 | 18,185 | 1,9991 | *** |
| C16:0 | 86,133 | 173,239 | 63,033 | 204,485 | 2,0113 | *** |
| C18:0 | 29,308 | 35,802 | 11,208 | 23,668 | 1,2216 | ns |
| C20:0 | 1,073 | 1,667 | 0,618 | 1,365 | 1,5533 | ** |
| C22:0 | 0,895 | 1,255 | 0,611 | 1,146 | 1,4028 | * |
| C24:0 | 1,073 | 1,770 | 1,452 | 2,836 | 1,6486 | * |
| C26:0 | 0,164 | 0,277 | 0,272 | 0,662 | 1,6922 | ns |
| eFAs | 127,23 | 229,95 | 76,614 | 242,747 | 1,8074 | ** |
| C13:0 | 0,176 | 0,274 | 0,119 | 0,164 | 1,5532 | *** |
| C15:0 | 5,395 | 11,509 | 4,703 | 14,226 | 2,1333 | *** |
| C17:0 | 1,942 | 3,641 | 1,506 | 3,557 | 1,8750 | *** |
| C19:0 | 0,320 | 0,509 | 0,226 | 0,426 | 1,5939 | ** |
| C21:0 | 0,634 | 0,747 | 0,886 | 1,037 | 1,1783 | ns |
| C23:0 | 0,527 | 0,721 | 0,332 | 0,698 | 1,3668 | * |
| C25:0 | 0,526 | 0,605 | 0,391 | 0,546 | 1,1487 | ns |
| oFAs | 9,520 | 18,006 | 6,659 | 18,927 | 1,8913 | *** |

| **Cheek (µg)** | **NA** | **A** | SD NA | SD A | FC | Significance |
| --- | --- | --- | --- | --- | --- | --- |
| C14:1 | 0,310 | 0,545 | 0,280 | 0,583 | 1,7583 | *** |
| C15:1 | 0,306 | 0,623 | 0,263 | 0,699 | 2,0341 | **** |
| C16:1 | 7,528 | 19,506 | 9,105 | 43,911 | 2,5910 | *** |
| C17:1 | 1,038 | 2,257 | 0,920 | 3,070 | 2,1746 | *** |
| 14Me-C17:1 | 0,197 | 0,389 | 0,168 | 0,402 | 1,9718 | *** |
| 15Me-C17:1 | 0,080 | 0,173 | 0,055 | 0,148 | 2,1687 | *** |
| C18:1 | 16,309 | 32,234 | 11,508 | 45,915 | 1,9765 | * |
| C20:1 | 0,439 | 0,731 | 0,321 | 0,692 | 1,6657 | * |
| C22:1 | 0,055 | 0,092 | 0,073 | 0,135 | 1,6766 | * |
| C24:1 | 0,112 | 0,171 | 0,138 | 0,247 | 1,5329 | * |
| MUFAs | 26,37 | 56,72 | 22,200 | 94,460 | 2,1506 | *** |
| C18:2 | 2,437 | 2,961 | 1,609 | 1,553 | 1,2149 | * |
| C20:2 | 0,537 | 0,511 | 0,347 | 0,289 | 0,9501 | ns |
| PUFAs | 2,974 | 3,471 | 1,628 | 1,583 | 1,1671 | * |
| FFAs | 341,88 | 632,27 | 216,355 | 728,927 | 1,8494 | *** |
| FOHC14:0 | 0,220 | 0,265 | 0,104 | 0,106 | 1,2053 | * |
| FOHC16:0 | 4,739 | 7,011 | 8,288 | 14,065 | 1,4794 | ns |
| FOHC18:0 | 18,255 | 23,228 | 31,588 | 33,272 | 1,2724 | ns |
| FOHC20:0 | 2,253 | 2,619 | 0,819 | 0,815 | 1,1623 | * |
| FOHC22:0 | 2,318 | 2,633 | 0,712 | 0,743 | 1,1363 | * |
| FOHC24:0 | 0,926 | 1,068 | 0,352 | 0,531 | 1,1525 | ns |
| FOHC26:0 | 0,543 | 0,621 | 0,402 | 0,549 | 1,1439 | ns |
| FOHs | 29,255 | 37,446 | 36,873 | 42,378 | 1,2800 | * |

| **Cheek (µg)** | **NA** | **A** | SD NA | SD A | FC | Significance |
| --- | --- | --- | --- | --- | --- | --- |
| Vitamin E | 0,081 | 0,104 | 0,062 | 0,112 | 1,2890 | ns |
| Cholesterol | 16,221 | 19,008 | 5,209 | 6,067 | 1,1719 | ns |
| Squalene | 177,05 | 340,66 | 116,383 | 173,777 | 1,9241 | **** |
| TGs | 260,84 | 386,24 | 122,831 | 124,479 | 1,4807 | **** |
| WEs | 187,12 | 261,40 | 82,902 | 83,898 | 1,3970 | **** |
| CEs | 133,95 | 134,27 | 27,236 | 21,805 | 1,0024 | ns |
| Sebum | 1146,3 | 1811,3 | 449,72 | 817,34 | 1,5801 | **** |
| C16:1/C16:0 | 7,618 | 8,690 | 2,720 | 3,465 | 1,1408 | ns |
| C17:1/C17:0 | 50,543 | 55,072 | 11,534 | 12,257 | 1,0896 | ns |
| C18:1/C18:0 | 55,647 | 79,698 | 29,096 | 47,770 | 1,4322 | *** |

FC, fold change of A vs NA

ns, not-significant

*p≤0.05, **p<0.001, ***p<0.005, ****p<0.0001 (Mann-Whitney test)
