# Supplementary material for: Lipidomics of facial sebum in the comparison between acne and non-acne adolescents with dark skin
Source: Sci Rep. 2021 Aug 16;11:16591. doi: 10.1038/s41598-021-96043-x (PMC8367971; doi:10.1038/s41598-021-96043-x)
Supplement: Supplementary file 8 — Supplementary Legends. [file 41598_2021_96043_MOESM8_ESM.docx]

Supporting information

Appendix

Supplementary Figure S1

Supplementary Figure S2

Supplementary Figure S3

Supplementary Table S1

Supplementary Table S2

Supplementary Table S3

Legend to Supplementary Figures

Supplementary Figure S1. Representative chromatograms of sebum analyzed by HPTLC (A) and GCMS (B1). The elution of squalene, cholesterol esters (CEs), wax esters (WEs), triglycerides (TGs), free fatty acids (FFAs), and cholesterol in the mixture of authentic standards are marked on the side of the representative HPTLC analysis (A). Each plate was loaded with extracts of blank tubes, four dilution levels of the standard mixture, a quality control (QC) sample, 12 sebum extracts from foreheads and cheeks of three acne (A) and non-acne (NA) donors, and with the extract of blank tapes, in the order.

Peaks of internal standards (red) and representative individual lipid molecules (black) are labelled with their correspondent name in the total ion current (TIC) chromatogram acquired by GCMS (B1). The extracted ion chromatograms (EIC) below the TIC (B2) correspond to the isomers of FA C16:0 (EIC 313), to the isomers of FA C16:1 (EIC 311) and to the deuterated FA C16:0 (EIC 330). The portion of the TIC between 16.8 and 17.9 minutes illustrates the GCMS elution of isobaric FFAs and FOHs (B3). The isomers of FA C15:0 and the isobaric FOH C16:0 are depicted in the EIC of the 299 m/z (EIC 299) below the TIC in the same retention time window.

Supplementary Figure S2. Radar diagrams depicting the distribution of absolute amounts (µg) of FFAs and FOHs within subclasses in acne (A) and non-acne (NA) groups.

Supplementary Figure S3. Sebum excretion rates (SER) calculated from sebum quantified in samples collected on foreheads and cheeks in unaffected (NA) and affected (A) groups. The green line connects the average values for each subgroup.
